# The Jellyfish *Rhizostoma pulmo* (Cnidaria): Biochemical Composition of Ovaries and Antibacterial Lysozyme-like Activity of the Oocyte Lysate

**DOI:** 10.3390/md17010017

**Published:** 2018-12-29

**Authors:** Loredana Stabili, Lucia Rizzo, Francesco Paolo Fanizzi, Federica Angilè, Laura Del Coco, Chiara Roberta Girelli, Silvia Lomartire, Stefano Piraino, Lorena Basso

**Affiliations:** 1 Department of Biological and Environmental Sciences and Technologies, University of Salento, Via Prov.le Lecce Monteroni, 73100 Lecce, Italy; fp.fanizzi@unisalento.it (F.P.F.); federica.angile@unisalento.it (F.A.); laura.delcoco@unisalento.it (L.D.C.); chiara.girelli@unisalento.it (C.R.G.); silvia_03_@hotmail.it (S.L.); 2 Water Research Institute (IRSA) of the National Research Council, S.S. Talassografico of Taranto, Via Roma 3, 74122 Taranto, Italy; 3 Consorzio Nazionale Interuniversitario per le Scienze del Mare, CoNISMa, Piazzale Flaminio 00196, 9- Roma, Italy; lucia.rizzo@szn.it; 4 Integrative Marine Ecology, Stazione Zoologica Anton Dohrn, Villa Comunale, 80121 Napoli, Italy

**Keywords:** antibacterial activity, NMR spectroscopy, biochemical characterization, jellyfish blooms

## Abstract

Jellyfish outbreaks in marine coastal areas represent an emergent problem worldwide, with negative consequences on human activities and ecosystem functioning. However, potential positive effects of jellyfish biomass proliferation may be envisaged as a natural source of bioactive compounds of pharmaceutical interest. We investigated the biochemical composition of mature female gonads and lysozyme antibacterial activity of oocytes in the Mediterranean barrel jellyfish *Rhizostoma pulmo*. Chemical characterization was performed by means of multinuclear and multidimensional NMR spectroscopy. The ovaries of *R. pulmo* were mainly composed of water (93.7 ± 1.9% of wet weight), with organic matter (OM) and dry weight made respectively of proteins (761.76 ± 25.11 µg mg^−1^ and 45.7 ± 1.5%), lipids (192.17 ± 10.56 µg mg^−1^ and 9.6 ± 0.6%), and carbohydrates (59.66 ± 2.72 µg mg^−1^ and 3.7 ± 0.3%). The aqueous extract of *R. pulmo* gonads contained free amino acids, organic acids, and derivatives; the lipid extract was composed of triglycerides (TG), polyunsaturated fatty acids (PUFAs), diunsaturated fatty acids (DUFAs), monounsaturated fatty acids (MUFAs), saturated fatty acids (SFAs), and minor components such as sterols and phospholipids. The *R. pulmo* oocyte lysate exhibited an antibacterial lysozyme-like activity (mean diameter of lysis of 9.33 ± 0.32 mm corresponding to 1.21 mg/mL of hen egg-white lysozyme). The occurrence of defense molecules is a crucial mechanism to grant healthy development of mature eggs and fertilized embryos (and the reproductive success of the species) by preventing marine bacterial overgrowth. As a corollary, these results call for future investigations for an exploitation of *R. pulmo* biomasses as a resource of bioactive metabolites of biotechnological importance including pharmaceuticals and nutrition.

## 1. Introduction

Anthozoans and medusozoans (commonly known as polyps and jellyfish, respectively) belong to Cnidaria, a group of approximately 10,000 marine invertebrates known to produce complex proteinaceous venomous mixtures used for defense and prey capture and delivered through highly specialized, epithelial mechano-sensor cells (the cnidocytes). A relatively small number of jellyfish (including scyphozoans, cubozoans, and hydrozoans) exhibit life history traits promoting reproductive success and formation of large aggregated populations [1]. Their regular outbreaks represent an emergent problem in worldwide coastal areas, favored by the rise of sea surface temperatures [2,3,4,5].

Routinely exposed to marine microbes, including viruses, bacteria, protists, and parasites, cnidarians seem unaffected by attacks of pathogens [6] even in the absence of typical protection systems of other metazoans (e.g., cuticle, hemolymph, phagocytic cells). Nevertheless, cnidarians possess a repertory of defense mechanisms, including the production of bioactive compounds involved in the recognition and neutralization of invaders [7]. In many cnidarians, sexual reproduction requires external fertilization, and the presence of defense molecules is crucial in the environment to secure protection of eggs and embryos, which contain energy-rich materials, against eukaryotic predators and bacteria [8]. Mortality rates of marine invertebrate eggs and larvae are high due to predation and diseases caused by marine microorganisms [9]. However, gametes of several invertebrates are equipped with several defensive substances, including IgM-like molecules [10], lectins [11], and antifungal and antibacterial proteins [12]. In *Hydra*, for instance, embryos are defended by a maternally produced antimicrobial peptide (AMP) of the periculin peptide family, which controls microbial colonizers during embryogenesis [8]. The endogenous AMPs are among the most important effectors of invertebrate innate immunity. A wide variety of antimicrobial peptides has been extracted from sponges, annelids, mollusks, crustaceans, tunicates, and cnidarians, however, AMPs still remain a largely unexplored resource, representing the starting point for the development of new antibiotics with a natural broad spectrum of action [13]. Some of these compounds do not easily allow bacteria to develop resistance towards them [14]. Over the recent decades, the metabolomics profiling and biochemical evaluation of a number of cnidarian species led to the discovery of more than 2000 natural compounds with antimicrobial/antibiotic properties [15]. 

Among the antimicrobial enzymes, lysozyme is the best characterized lytic agent, capable of breaking the peptidoglycan-based bacterial cell wall, causing high-pressure osmotic cytolysis and burst [16]. Several methods are available to measure lysozyme activity, including the spectrophotometric method and the standard assay on inoculated Petri dishes [17,18]. Lysozyme widely occurs in marine protostomes and deuterostomes, including polychaetes and echinoderms [19], particularly in secretions such as mucus. Recently, an antibacterial lysozyme-like activity was found in the anthozoan *Actinia equina* mucus [20], presumably with a defensive role against potential predation by the surrounding microorganisms. In this framework, the so-called “white barrel” or “sea lung” *Rhizostoma pulmo* (Scyphozoa) is one of the largest and most abundant jellyfishes along the Mediterranean coasts. The medusa stage of this species is known to produce considerable amounts of sticky mucus used to either entrap food particles or as a deterrent against predators. The large biomass reached by its populations along the Mediterranean coasts has recently suggested this species as a top candidate for isolation and sustainable production of bioactive compounds for pharmaceutical applications or for nutritional purposes [21]. In this framework, the biochemical composition and the metabolic profile (by ^1^H NMR spectroscopy) of *R. pulmo* ovaries as well as the antimicrobial properties of oocytes were investigated. 

## 2. Results

### 2.1. Biochemical Composition of Ovaries 

**Figure 1:**
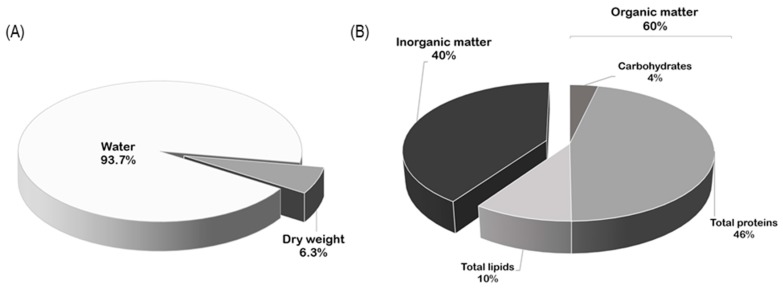
*Rhizostoma pulmo* ovaries composition: (**A**) water content and dried weight; (**B**) inorganic and organic residues.

The water content of *R. pulmo* ovaries was 93.7 ± 1.9% (Figure 1A). After dehydration, organic matter was the main part (60.43 ± 10.14%) of ovary dry weight (Figure 1B). The organic residue of the ovaries was mainly composed of proteins (761.76 ± 25.11 µg mg^−1^ OM), lipids (192.17 ± 10.56 µg mg^−1^ OM), and carbohydrates (59.66 ± 2.72 µg mg^−1^ OM); Figure 1B).

Among lipids, the mean triglyceride and cholesterol concentrations were 0.12 ± 0.008 mg/mL and 0.24 ± 0.0023 mg/mL, respectively. 

### 2.2. NMR Spectroscopy 

#### 2.2.1. NMR Analysis of Female Gonads Aqueous Extracts 

**Figure 2:**
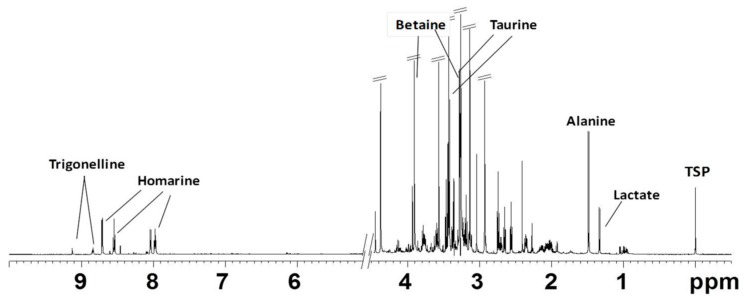
Typical ^1^H NMR spectrum obtained at 600 MHz of *R. pulmo* ovaries aqueous extract.

**Figure 3:**
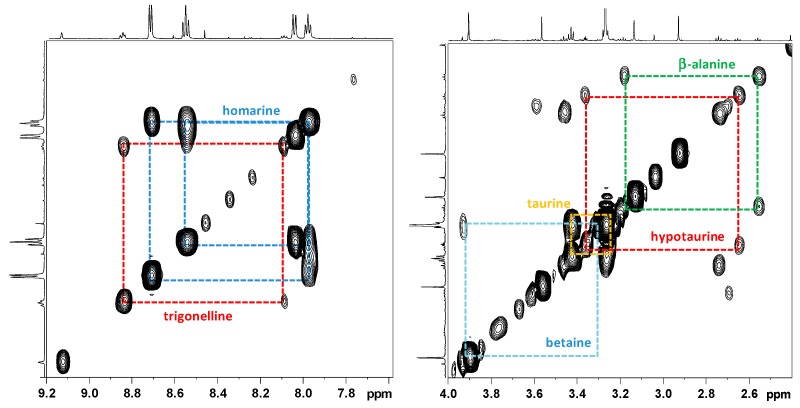
Expansions of COSY spectrum of *Rhizostoma pulmo* aqueous extract. Colored boxed regions correlate with the various resonances of homarine, trigonelline, betaine, taurine, hypotaurine, and glycine.

The ^1^H NMR spectrum of the aqueous extract of *R. pulmo* ovaries was characterized by free amino acids, organic acids, and derivatives (Figure 2). Many signals due to different compounds, such as betaine (δ 3.27 and 3.90), taurine (δ 3.27 and 3.41), homarine (δ 4.37, 7.97, 8.04, 8.55, 8.71), lactate (δ 1.33 and 4.16), succinate (δ 2.41), acetate (δ 1.92), and formate (δ 8.46), were identified. High signals at δ 3.57 and the doublet at δ 1.48 were also assigned to glycine and alanine, respectively. The multiplet of proline appeared at δ 2.1–2.0, 2.32–2.36, 3.30–3.40, 4.10–4.14, while the multiplet at δ 2.07 and 2.36 were assigned to glutamate. Other amino acids such as leucine (δ 0.96, 1.70), isoleucine (δ 1.01, 1.97), valine (δ 1.05, 2.29), threonine (overlapping doublets at δ 1.33 and multiplets at δ 3.68 and 4.29) were detected. Using 2D NMR experiments (Figure 3), and by comparison with literature data [22,23,24], two other osmolytes were identified: β-alanine (triplets at δ 2.56 and 3.20) and hypotaurine (triplets at δ 2.65 and 3.37). In the aromatic region, low-intensity signals at δ 6.90 and 7.19 were assigned to tyrosine, while δ 8.08, 8.84, and 9.13 signals were also identified for trigonelline (*N*-methylpicolinic acid). Quantitative analysis [25] showed that taurine, betaine, and glycine (concentrations >10 mM) were the most abundant free metabolites. Homarine, β-alanine, and alanine contents ranged from 5 to 3 mM. Finally, trigonelline, acetate, valine, formate, succinate, and hypotaurine were present in very low concentrations (≤1 mM).

#### 2.2.2. NMR Analysis of Female Gonads Lipid Extracts 

**Figure 4:**
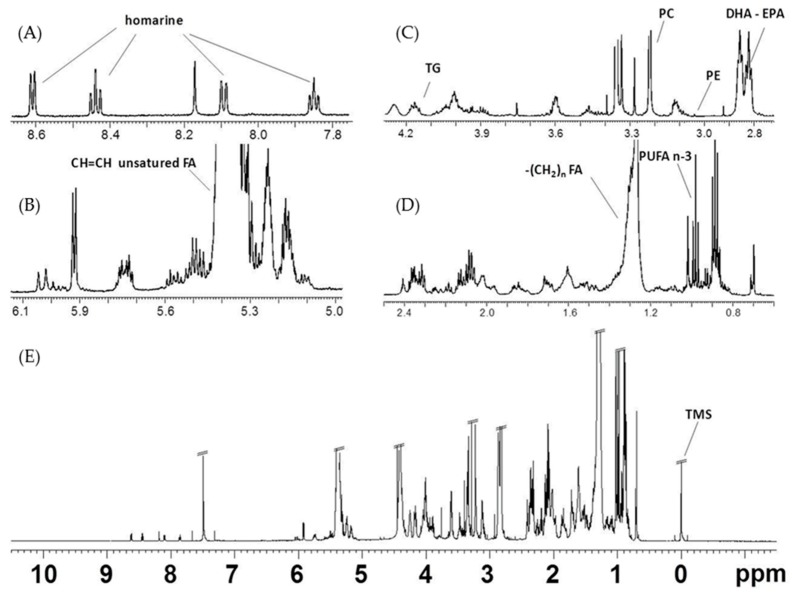
Typical ^1^H NMR spectrum obtained at 600MHz of CD_3_OD/CDCl_3_ *R. pulmo* female gonad lipid extract (**a**) high-, (**b**,**c**) middle-, and (**d**) low-frequency regions, (**e**) full spectrum.

The lipid extracts of the examined jellyfish female gonads were characterized by the presence of triglycerides (TG), polyunsaturated fatty acids (PUFAs), diunsaturated fatty acids (DUFAs), monounsaturated fatty acids (MUFAs), saturated fatty acids (SFAs), and minor components such as sterols (cholesterol) and phospholipids. The main signals, marked in the spectrum (Figure 4), corresponded to −CH_2_ in α and β-position to the carboxylic acid esters (COOCH_2_CH_2_), unsaturations (CH=CH-CH_2_-CH=CH), and monounsaturated fatty acids (docosahexaenoic, DHA C22:6, and eicosapentaenoic acids, EPA C20:5, ω-3) or other PUFAs (two and more than two double bonds) of long fatty acids alkyl chain, including PUFA CH_3_s and terminal CH_3_s of phospholipids. 

The COSY cross peaks correlated with the multiplets from the glycerol moiety of TG appeared at δ 4.14 and 4.11 (sn 1,3) and δ 5.24 (sn 2), with very low intensities. The signals in the range of δ 2.32–2.27 and δ 1.66–1.57 were assigned to protons of COOCH_2_ and COOCH_2_CH_2_, respectively, for all the fatty acids chains, except for DHA (signal at δ 2.38, COOCH_2_CH_2_,) and EPA (signal at δ 1.70 COOCH_2_CH_2_). 

**Figure 5:**
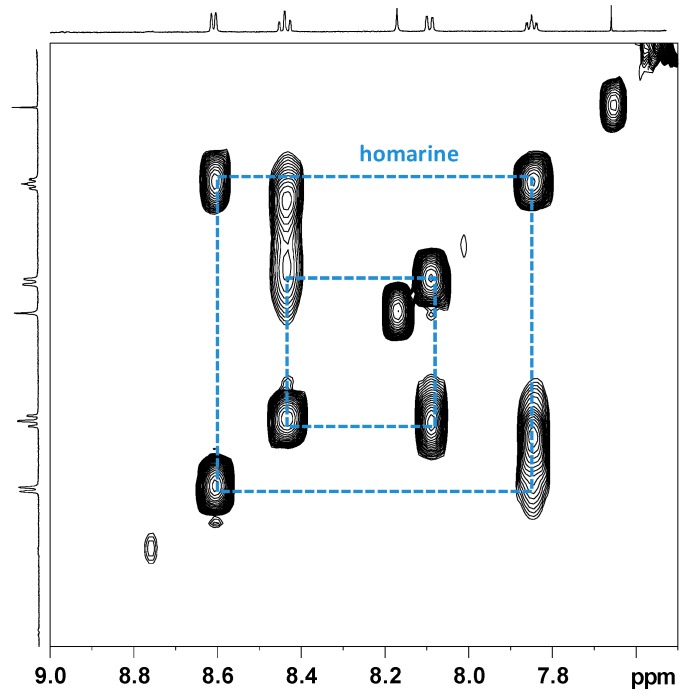
Expansion of the COSY spectrum of *R. pulmo* lipid extract. Colored boxed regions correlate with the various resonances of homarine.

The presence of ω-3 PUFAs is confirmed by the appearance of a triplet at δ 0.98 related to the terminal methyl group. This terminal methyl group is clearly separated from other methyl groups at δ 0.80 and 0.91, ascribable to all other non-ω-3 fatty acids, such as DUFAs, MUFAs, and SFAs. The spectra also indicated intense signals in the range δ 2.88–2.75 for the presence of bis-allylic (CH=CH-CH_2_-CH=CH) protons of long alkyl chain fatty acids components. In particular, the multiplet at δH 2.85–2.80 were assigned to bis-allylic protons (CH=CH-CH_2_-CH=CH) of PUFAs (such as DHA and EPA), while bis-allylic protons of other PUFAs, such as α-linolenic fatty acid and DUFAs, appeared at δH 2.77. The presence of partially overlapping singlets at δH 3.22 are due to the polar head group (N(CH_3_)_3_) of phosphatidylcholine (PC), while the signal at δH 3.03 is attributed to the CH_2_N group of phosphatidylethanolamine (PE). The presence of phospholipids in the extracts was confirmed by ^31^P NMR analysis of few samples (data not shown). Furthermore, signals at δH 0.68–0.69, 0.92, and 1.01, due to characteristic resonances of cholesterol (CHO) and multiplets in ranges of δH 5.26–5.13 and 4.28–4.12, assigned to 1,2-diacylglycerols (DAGs), were also observed. Finally, homarine signals appeared at δH 8.61, 8.44, 8.09, 7.85. The assignments were confirmed by 2D experiments (Figure 5) and literature data [26,27,28,29]. 

### 2.3. Lysozyme-like Activity in R. pulmo Oocyte Lysate 

**Figure 6:**
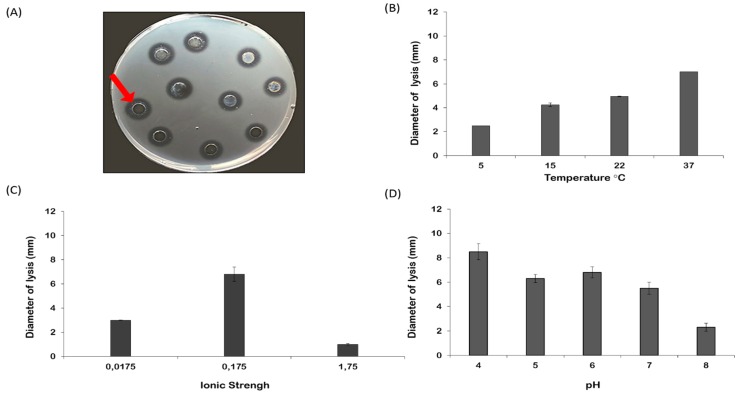
(**A**) Lysozyme-like activity of *R. pulmo* oocyte lysate measured on Petri dish. The arrow indicates the diameter of lysis around each well (6.3 mm in diameter) in which the oocyte lysate (30 μL) was loaded. All the wells were loaded with 30 μL of oocyte lysate and represent replicates; (**B**) effect of the temperature (5, 15, 22, and 37 °C) on lysozyme-like activity measured at ionic strength (I) = 0.175 and pH 6.0; (**C**) the effect of ionic strength (I = 0.0175, 0.175, 1.75) on lysozyme-like activity measured at temperature 37 °C and pH 6.0; (**D**) the effect of the pH (4, 5, 6, 7, 8) on lysozyme-like activity measured at 37 °C and I = 0.175. Data are reported as mean value ± standard error.

**Figure 7:**
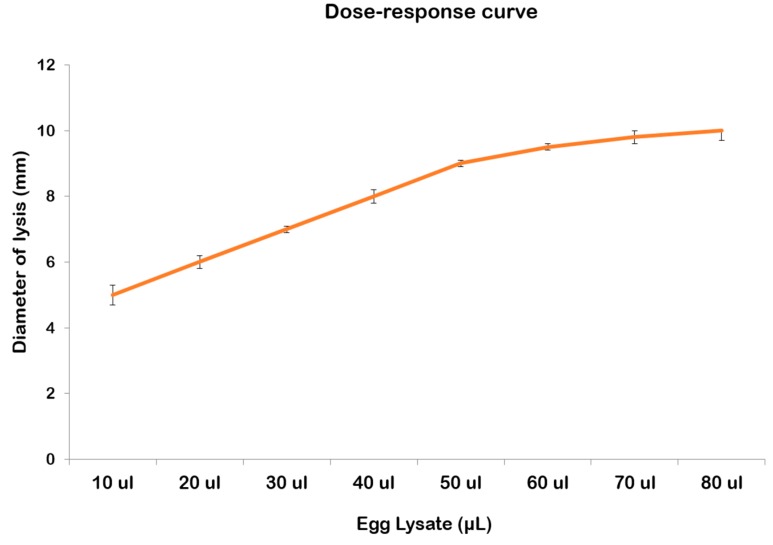
Dose-response curve of lysozyme-like activity of *R. pulmo* oocyte lysate.

Oocyte lysate of *R. pulmo* showed a natural lysozyme-like activity. By the standard assay on Petri dishes, a diameter of lysis of 9.33 ± 0.32 mm corresponding to 1.21 mg/mL of hen egg-white lysozyme was observed (Figure 6A). The lysozyme activity of the egg lysate was significantly affected by temperature (*p* = 0.0002), ionic strength (*p* = 0.0028), and pH (*p* = 0.0002) of the incubation (Figure 6B–D; Table 1). Post hoc analyses clarified better responses at different experimental conditions tested (Table 2). Increasing the temperature improved proportionally lysozyme-like activity (Figure 6B), showing significant differences among different conditions. The lytic activity increased significantly after dialysis against PB at I = 0.175 (Figure 6C, Table 2). Among all experiments, the maximum diameter of lysis was reported at pH 4.0, although there were no significant differences among the measured diameters at pH 4 or 6 (Figure 6D, Table 2). A dose-response correlation was obtained when increasing amounts of oocyte lysate were plotted against the respective lysis area diameters (Figure 7). The diameter of the lysis area was positively correlated with the sample volume.

## 3. Discussion

The apparent increase of global jellyfish abundance in coastal marine ecosystems has recently attracted scientific interest for the potential impacts on human activities and ecosystem functioning. Also, the possible use of jellyfish biomass as a source of energy and bioactive compounds useful for pharmaceutical and nutritional applications has been suggested [30,31]. In this context, understanding the biological mechanisms underlying jellyfish outbreaks is crucial to predict and/or mitigate impacts of recurrent bloom events. The occurrence of jellyfish outbreaks is also directly linked to the reproductive success. The present paper represents the first insight into the biochemical composition of ovaries and the lysozyme antibacterial activity associated with oocytes of the barrel jellyfish *R. pulmo* in order to investigate aspects related to the mechanisms boosting the success of sexual reproduction.

In *R. pulmo*, more than 90% of the ovary volume is composed of water in accordance with previous studies on *R. octopus* [32] and other scyphozoan jellyfish gonads (e.g., *Cyanea capillata*, *Chrysaora hysoscella*, and *Pelagia nocticula* [5,32]). The organic matter in *R. pulmo* ovaries represented 60% of dry weight and was composed mainly of proteins, lipids, and low content of carbohydrates, corroborating the general trend in gonadal composition recorded in other scyphozoans, including *P. nocticula* [5,32]. In particular, proteins were twofold concentrated in *R. pulmo* female gonads compared to *C. capillata* and threefold compared to *R. octopus* gonads [32]. The biochemical composition of *R. pulmo* ovaries reflects the composition of the entire jellyfish in which the organic content is mainly represented by protein, followed by lipid and carbohydrate fractions [21].

The ^1^H NMR characterization of both lipid and aqueous extracts of *R. pulmo* female gonads provided advanced information on the chemistry of this jellyfish compartment. As known [22,33], the untargeted ^1^H NMR-based metabolomic approach could be used to provide simultaneous determination of the end products of metabolism, such as small-molecular-weight molecules in solution [24]. In the present study, many ^1^H NMR signals of the aqueous extract were due to free amino acids, such as leucine, isoleucine, valine, threonine (essential amino acids) and alanine, glycine, proline, and glutamate, representing also the dominant amino acids in the gonads of the edible Asiatic jellyfish *Rhopilema esculentum* [34] and other edible jellyfish species [35]. In the aromatic region, ^1^H NMR analysis revealed also the presence of tyrosine, which is considered an essential amino acid for humans, useful during stages of human prenatal development [36]. Alanine, glycine, and glutamate are also implicated in other physiological processes and on account of these features are considered useful as antioxidant constituents in foodstuffs and beverages [37]. Thus, the *R. pulmo* ovaries could represent a source of amino acids exploitable for nutraceutical and pharmaceutical applications as well as a source of proteins for the development of innovative dietary supplements for fish nourishment. In this framework indeed, considering the growing cost of fish feed worldwide, each innovative natural resource as a potential ingredient in their preparation must be considered. Furthermore, the aqueous extract was characterized by the presence of compounds with important metabolic roles in marine invertebrates [23,38,39], such as the osmolytes trimethylamine *N-*oxide (TMAO), betaine, taurine, and homarine. Interestingly, in the colonial hydropolyp *Hydractinia echinata* (Hydrozoa), it was observed that betaine, homarine, and trigonelline, when present simultaneously, play a regulatory role during the development and prevent the onset of metamorphosis [40,41]. On account of the high amount evidenced in *R. pulmo* gonads, it is likely that, as in the case of *H. echinata*, also in the here-investigated jellyfish, these compounds could be involved in maintaining the larval state until an appropriate signal allows metamorphosis. Moreover, TMAO is an osmolyte that commonly occurs in several marine animals and it has been found to neutralize the effects of hydrostatic pressure on cnidarian, fish, and mammalian [42]. The densities differ widely among habitats, species, and season and ontogeny within species A relationship exists between the concentration of TMAO (and betaine) in muscle tissue and lipid. At present, we do not rule out the possibility that, in *R. pulmo* ovaries, TMAO plays a role for its protein-stabilizing attributes as already hypothesized for several marine organisms [43].

In the female gonads of *R. pulmo*, a higher content of total lipids was also recorded in comparison with the total jellyfish [21]. As reported in literature [21,44], the lipid composition of jellyfish can be considerably influenced by several external factors such as diet, size, and age of organism. The lipid NMR analysis of *R. pulmo* gonads showed the presence of different lipid classes. As already observed in the whole jellyfish [21], the analysis of *R. pulmo* ovaries confirmed the presence of a high content of ω-3 PUFAs. In the gonads of marine invertebrates, there is a notable richness of PUFAs, particularly 20:5 ω-3 and 22:6 ω-3 [45]. PUFAs actively participate in gonad maturation, egg quality [46], and larval growth of fish [47]. In the common octopus, *Octopus vulgaris*, PUFAs can improve membrane fluidity and flexibility of spermatozoa membrane, and are actively implicated in the regulation of cellular movement, gonadal metabolism of lipids, and fusion capacity [48]. In crustaceans and mollusks, PUFAs not only determine hatching and growth [49] but also play an important role in metabolism processes, like production of prostaglandins and hormones, and regulate ionic fluxes [50]. Among the categories of fatty acids (FAs) of *R. pulmo* ovaries, the two signals of EPA and DHA were also revealed. Noteworthy, these categories of fatty acids were already recorded in other jellyfish, including *Aurelia* sp., whose fatty acid profiles were broadly similar to 16:0, EPA, 18:0, AA, and DHA as the five main components accounting for around 66% of the total FAs. Furthermore, EPA and DHA have been detected in different species of *Aurelia* jellyfish [44,51]. The presence of ω-3 PUFAs, mainly DHA and EPA, in the gonads of *R. pulmo* suggests their potential exploitation as sources of these compounds for the application in the pharmaceutical field. It is well known indeed that ω-3 PUFAs, DHA and EPA, possess antioxidant and anti-inflammatory properties useful for potential treatment strategies for mental health and neuro-inflammation-induced memory deficits [52,53]. Moreover, taking into account that diets for fish are usually enriched with additional supplements of EPA and DHA, the gonads of *R. pulmo* could furnish these essential FAs to be added in the production of fish feed.

An antibacterial lysozyme-like activity was detected in the oocyte lysate of *R. pulmo*. As in the majority of scyphozoans, also in *R. pulmo*, reproduction is external; thus, an antibacterial activity may prevent eggs and embryos from being overgrown and killed by pathogenic bacteria. To our knowledge, this is the first record of an antimicrobial activity in jellyfish eggs. Many marine taxa synthesize specific metabolites to protect themselves against the settlement and growth of microbial agents. For example, surface attachment and growth of several marine bacteria are inhibited by the extracts from the eggs of several coral species [54]; potentially pathogenic bacteria, including a *Vibrio* sp., are subjected to the toxic action induced by the extracts of various developmental stages of the soft coral, *Parerythropodum fulvum* [55]. Gunthorpe and Cameron [56,57] found that in some species of soft corals, the extracts exerted an antibiotic activity negatively related to the presence of immature gonads, suggesting that reproductive status represented a significant predictor of antimicrobial activity [56]. A similar phenomenon was found in the octocoral *Lobophytum compactum*; indeed, antimicrobial diterpenes were selectively included into egg lipid material [58] detectable in adults before spawning and absent afterwards. Moreover, the extracts of the damselfish *Pomacentrus mollucensis* eggs is defended chemically [59]. In addition, egg extracts of the coral species *Montipora digitata* are able to produce growth inhibition of *Escherischia coli* [60]. Among marine invertebrates, also in echinoderms, characterized by external fertilization, the eggs and larvae from the regular echinoid *Paracentrotus lividus* exert an antibacterial lysozyme-like activity [16]. Lysozyme-like proteins have also already been evidenced in other cnidarians [61,62]. Regarded as the best and most active enzyme involved in the innate immunity [63], lysozyme is a glycoside hydrolase whose constitutive levels defend the organism from pathogenic bacteria present in the surrounding environment and regulate natural symbiotic microflora. Besides antimicrobial activity, lysozymes play a role in digestion, antiviral, anti-inflammatory, and antitumor activities, taking part in the innate immunity as first defensive line [64]. It is well known that lysozyme activity is affected by various factors such as temperature, pH, and salts [65,66]. In the case of *R. pulmo* egg lysate, the highest lysozyme-like activity is detected at pH 4 and the ionic strength 0.175. A similar result was already obtained from egg lysate of the sea star *Marhasterias glacialis* (maximum of activity at pH 4.2 and the ionic strength 0.175) [16]. Further studies will be undertaken to assess whether the category of lysozyme involved in egg protection is the same in marine invertebrates as well as to estimate the antibacterial activity against other living microorganisms besides *Micrococcus luteus.* At this stage, we only focused on the occurrence of a lysozyme-like activity in the whole oocyte lysate. However, to further elucidate the mechanisms related to *R. pulmo* egg defenses, it is necessary to perform the isolation, purification, and quantification of the effectors of such antimicrobial activity which will be carried out in the near future. 

The evidence of a lysozyme-like activity in *R. pulmo* oocyte lysate suggests that this species may also represent a new and exciting resource for the extraction of potent antibacterial agents and encourages the potential use of the jellyfish for lysozyme-based preparations in pharmaceutical research. Currently, lysozyme is used for pharmaceutical preparations due to the therapeutic effectiveness of lysozyme based not only on its ability to control the growth of bacteria but also to modulate the immune responses of the host. Moreover, the treatment with lysozyme leads to a regression in the growth of some tumor cells [67]. Lysozyme can be also used in the treatment of a wide range of infections in humans since it has no toxic effect on humans and thus it is a good candidate for the use of epidermal and cosmetic formulations. Finally, considering that controlling bacterial infections is currently one of the main problems of aquaculture on an industrial scale, lysozyme is attracting the interest of researchers for its potential applications in aquaculture. 

## 4. Materials and Methods 

### 4.1. Sample Collection and Preparation

Sixty specimens of *R. pulmo* adult medusae (umbrella diameter > 25 cm at sexual maturity; Basso, personal observations) were sampled at the Ginosa Marina (Ionian Sea 40°25.7′ N, 16°53.1′ E; Italy) throughout 2017 with a 1 cm mesh hand net from a boat. Immediately after sampling, jellyfish were transported into the laboratory and washed with filter-sterilized seawater (0.2 μm, Millipore) to remove the mucus layer produced during transport. Ovaries appeared from pink to orange, with easily distinguishable eggs. When gender determination was uncertain visually, a small piece of gonad tissue was removed and examined under the stereomicroscope. The ovaries were carefully dissected with microscissors at the stereomicroscope to avoid loss of gonadic tissue or accidental inclusion of subumbrellar or exumbrellar tissues and mature eggs were collected from a number of mature gonads. Each gonad was then divided in two aliquots. The first aliquot was frozen at −80 °C in liquid nitrogen to be lyophilized and then employed for the biochemical and NMR analyses; the second aliquot was employed to obtain the mature eggs. In particular, the eggs were obtained in pasteurized seawater (PSW) by placing ovaries on four layers of gauze. Eggs were allowed to settle and, after removal of the supernatant, were resuspended in sufficient PSW to obtain a 10% (*v*/*v*) suspension. After that, the eggs were gently swirled and then centrifuged at 12,000 g for 30 min [68]. The resulting supernatant (oocyte lysate) was dialyzed against distilled water, then lyophilized, and then concentrated 10-fold in PSW and used to evaluate the lysozyme-like antibacterial activity.

### 4.2. Biochemical Analysis

The ovary organic matter content of *R. pulmo* was evaluated using approximately 10 mg (±0.01 mg) of dry tissue reduced to ash for 4 h at 500 °C in a muffle furnace (BICASA B.E. 34). This content was expressed as the percentage of organic matter of total tissue dry weight (DW)). The weight of organic matter (OM) was determined as the difference between the gonad DW and the ash weight [69]. Female gonads biochemical composition was performed in order to detect contents in protein, carbohydrate, and total lipid (*n* = 15). Ovary tissue was frozen in liquid nitrogen, temporarily stored at −20 °C, and briefly transferred one hour before lyophylisation to −80 °C to facilitate freeze-drying (48 h) for the biochemical and NMR analyses. 

Carbohydrate, protein, and lipid quantification was performed by colorimetric determination at 480 nm, 750 nm, and 520 nm, respectively. In order to calculate the carbohydrate content in the ovarian tissue, approximately 10 mg (±0.1 mg) of each lyophilized sample was homogenized in 3 mL of double distilled water [70] with glucose as a standard. The content of proteins was estimated by employing approximately 10 mg (±0.1 mg) of each lyophilized tissue sample homogenized in 2 mL of 1N NaOH [71] with albumin as a standard. Finally, total lipids were determined by homogenizing approximately 10 mg (±0.1 mg) of each lyophilized tissue sample in 3 mL of chloroform–methanol (2:1) with cholesterol as a standard [72]. Quantities were expressed as µg mg^−1^ of OM.

Cholesterol content was evaluated by homogenizing approximately 150 mg (±0.1 mg) of each lyophilized sample in 4 mL of distilled water and was calculated by the colorimetric enzymatic method using the commercial kit (10028 Cholesterol, SGM, Rome, Italy) based on Jacobs et al. [73] with known amounts of cholesterol standard. Finally, triglycerides were estimated by homogenizing approximately 150 mg (±0.1 mg) of each lyophilized tissue sample in 4 mL of distilled water and were measured by the colorimetric enzymatic method using the commercial kit (10160 Triglycerides, SGM, Rome, Italy) based on Bucolo and David [74].

### 4.3. NMR Analysis

Samples were prepared according to a modified Bligh and Dyer extraction method [75,76]. Lyophilized gonads (~100 mg) were added to 400 μL methanol, 400 μL deionized filtered water, and 400 μL chloroform. The solution was mixed and placed on ice for 10 min before centrifugation at 10000 rpm for 20 min at 4 °C. The polar and lipophilic phases were separated and dried by a SpeedVac concentrator (SC 100, Savant, Ramsey MN, USA). The lipid extracts were dissolved in 700 μL of CD_3_OD/CDCl_3_ (1:2 mix) containing 0.03% *v*/*v* tetramethylsilane (TMS, δ = 0.00) as internal standard. The aqueous extracts were dissolved in 160 μL 0.2 M Na_2_HPO_4_/NaH_2_PO_4_ buffer (pH 7.4) and 540 μL D_2_O containing 3-(trimethylsilyl)-propionic-2,2,3,3-d4 acid (TSP δ = 0.00) as internal standard). Both extracts were transferred into 5 mm NMR tubes for NMR analyses. 

#### NMR Spectroscopy and Data Processing

NMR spectra were obtained using a Bruker Avance III 600 Ascend NMR spectrometer (Bruker, Ettliger, Germany) operating at 606.13 MHz for ^1^H observation with a z axis gradient coil and automatic tuning-matching (ATM). Experiments were performed at 300 K in automation mode after loading individual samples on a Bruker Automatic Sample Changer, interfaced with the software IconNMR (Bruker). For aqueous extracts, a one-dimensional zgcppr Bruker standard pulse sequence was applied to suppress the residual water signal. A number of 32 repetitions of scans (with 4 dummy scans) were collected into 64 k data points with relaxation delay set to 5 s, a spectral width of δ 20.0276 (12,019.230 Hz), 90° pulse of 11.11 µs. For lipid extracts, a one-dimensional zg Bruker standard pulse sequence was acquired, with repetitions of 64 scans (with 4 dummy scans), a spectral width of δ 20.0276 (12,019.23 Hz) and 90° pulse of 10 μs. For both aqueous and lipid extracts, the FIDs were multiplied by an exponential weighting function corresponding to a line broadening of 0.3 Hz before Fourier transformation, phasing and baseline correction. Metabolites identified by the ^1^H NMR spectra were assigned on the basis of analysis of 2D NMR spectral analysis (2D ^1^H J *res*, COSY, HSQC, and HMBC) and by comparison with literature data [23,28,40]. Whenever possible, also quantitative analysis of free metabolites was determined by integrating selected unbiased NMR signals, using TSP for chemical shift calibration and quantification [25]. In particular, signals corresponding to valine (δ 1.05), alanine (δ 1.48), acetate (δ 1.92), succinate (δ 2.41), β-alanine, (δ 2.56), hypotaurine (δ 2.65), betaine (δ 3.90), taurine (δ 3.41), glycine (δ 3.57), homarine (δ 8.04), formate (δ 8.46), and trigonelline (δ 9.13) were integrated. All chemical reagents for analysis were of analytical grade. D_2_O, CDCl_3_, CD_3_OD (99.8 atom%D), TSP, 3-(trimethylsilyl)-propionic-2,2,3,3,d4 acid, and tetramethylsilane, TMS (0.03 *v*/*v*%) were purchased from Armar Chemicals (Döttingen, Switzerland).

### 4.4. Lysozyme-Like Activity

Normally, a spectrophotometric method is used to demonstrate the occurrence of lysozyme activity [17]; however, the standard assay on inoculated Petri dishes can be used as an alternative method to demonstrate the occurrence of lysozyme activity [18]. Here, the presence of lysozyme activity was detected by the standard assay on Petri dishes, which resulted as a quick, sensitive, low-cost, and therefore very versatile method [16,18,20,77,78,79]. Briefly, 700 μL of 5 mg/mL of dried *Micrococcus luteus* cell walls (Sigma, Saint Louis, MO, USA) were suspended in 7 mL of 0.05 M PB-agarose (1.2%, pH 5.0) then spread on a Petri dish. Four wells of 6.3 mm diameters were sunk in agarose gel and each filled with 30 μL of sample (oocyte lysate). After overnight incubation at 37 °C, the diameter of the cleared zone of at least four replicates was measured. Diameters of lysis were compared with those of reference obtained with known amounts of standard hen egg-white lysozyme (Merck, Darmstadt, Germany). The effects of pH, ionic strength (I), and temperature were assessed for each sample. The pH effect was tested by dialyzing (7000-MW cut-off), the samples in PB 0.05 M, ionic strength, I = 0.175, adjusted at pH 4, 5, 6, 7, 8, and by dissolving agarose in PB at the same I and pH values. The ionic strength effect was tested in PB 0.05 M (pH 6.0), adjusted at I = 0.0175, 0.175, 1.75. Agarose was dissolved in PB at the same I values. The temperature effect was evaluated by performing the Petri dish assays (in PB, at pH 6.0, and I = 0.175) and incubating the plates at 5, 15, 22, and 37 °C. The dose-response curve of lysozyme-like activity was constructed by using Petri dish assays (in PB, at pH 6.0, and I = 0.175) with different amounts of sample (10, 20, 30, 40, 50, 60, or 80 μL of sample in each well in triplicate). 

### 4.5. Statistical Analysis 

To test the effects of temperature, pH, and ionic strength on antibacterial activity of oocyte lysate, one-way permutational multivariate analyses of variance (PERMANOVA) [80] were performed based on Euclidean distances on untransformed data, using 9999 random permutations of the appropriate units [81], following three different designs with one factor, separately: temperature (Te, as fixed factor with four levels); pH (pH, as fixed factor with five levels); ionic strength (IS, as fixed factor with three levels). When significant differences were found (*p* < 0.05), post hoc pairwise tests were carried out to ascertain the consistency of the differences among several different conditions tested. Because of the restricted number of unique permutations in the pairwise tests, p values were obtained from Monte Carlo samplings. The analyses were performed using the software PRIMER v. 6 [82].

## 5. Conclusions

In conclusion, *R. pulmo* ovaries and oocytes could represent a promising source of bioactive compounds for different applications mainly in the pharmaceutical field or as specialty feed. In particular, the antibacterial lysozyme-like activity suggests that this jellyfish species may represent a new and renewable resource for drugs discovery. Moreover, the presence of ω-3 PUFAs encourages their potential exploitation as sources of these compounds in the production of fish feed. Further studies will help to standardize a sustainable exploitation pilot system to use different jellyfish fractions for different purposes (e.g., food, feed) and beneficial services for human wellbeing.

## Figures and Tables

**Table 1:** Results from the multivariate permutational analysis (PERMANOVA) showing differences in lysozyme activity among different tested conditions.

	df	MS	F	*p*
**Temperature** Residual Total	3 8 11	10.416 0.0163	640.72	***
**Ionic Strength** Residual Total	2 6 8	27.417 0.33799	81.117	**
**pH** Residual Total	4 10 14	21.35 0.58333	36.6	***

df = degree of freedom; MS = mean sum of squares; F = F value by permutation; *p* = *p*-value by permutation. ** = *p* < 0.01; *** = *p* < 0.001.

**Table 2:** Results of the pairwise tests showing differences in lysozyme activity among various levels in different laboratory conditions (temperature, ionic strength, pH).

Temperature	T	P(MC)	Ionic Strength	T	P(MC)	pH	T	P(MC)
5 vs. 15	12.12	***	0.0175 vs. 0.175	6.88	0.0029	4 vs. 5	4.025	*
5 vs. 22	84.87	***	0.0175 vs. 1.75	28.73	0.0001	4 vs. 6	2.646	ns
5 vs. 37	1561	***	0.175 vs. 1.75	10.21	0.0006	4 vs. 7	5.277	**
15 vs. 22	4.756	**				4 vs. 8	11	***
15 vs. 37	19.08	***				5 vs. 6	1	ns
22 vs. 37	70.83	***				5 vs. 7	1.89	ns
						5 vs. 8	13	***
						6 vs. 7	2.324	ns
						6 vs. 8	8.66	**
						7 vs. 8	12.12	***

T = T value; P(MC) = probability level after Monte Carlo simulations. * = *p* < 0.05; ** = *p* < 0.01; *** = *p* < 0.001; ns = not significant.

## References

[1] Hamner W.M., Dawson M.N. (2009). A review and synthesis on the systematics and evolution of jellyfish blooms: Advantageous aggregations and adaptive assemblages. Hydrobiologia.

[2] Falkenhaug T. (2014). Review of Jellyfish Blooms in the Mediterranean and Black Sea. Mar. Biol. Res..

[3] Benedetti-Cecchi L., Canepa A., Fuentes V., Tamburello L., Purcell J.E., Piraino S., Roberts J., Boero F., Halpin P. (2015). Deterministic factors overwhelm stochastic environmental fluctuations as drivers of jellyfish outbreaks. PLoS ONE.

[4] Boero F., Brotz L., Gibbons M.J., Piraino S., Zampardi S. (2016). Impacts and effects of ocean warming on jellyfish. Explaining Ocean Warming: Causes, Scale, Effects and Consequences.

[5] Milisenda G., Martinez-Quintana A., Fuentes V.L., Bosch-Belmar M., Aglieri G., Boero R.R., Piraino S. (2018). Reproductive and bloom patterns of Pelagia noctiluca in the Strait of Messina, Italy. Estuar. Coast. Shelf Sci..

[6] Bosch T.C.G. (2008). The Path Less Explored: Innate Immune Reactions in Cnidarians. Innate Immunity of Plants, Animals, and Humans.

[7] Mariottini G.L., Grice I.D. (2016). Antimicrobials from cnidarians. A new perspective for anti-infective therapy?. Mar. Drugs.

[8] Jung S., Dingley A.J., Augustin R., Anton-Erxleben F., Stanisak M., Gelhaus C., Gutsmann T., Hammer M.U., Podschun R., Bonvin A.M.J.J. (2009). Hydramacin-1, structure and antibacterial activity of a protein from the basal metazoan hydra. J. Biol. Chem..

[9] Rumrill S.S. (1990). Natural mortality of marine invertebrate larvae. Ophelia.

[10] Fuda H., Hara A., Yamazaki F., Kobayashi K. (1992). A peculiar immunoglobulin M (IgM) identified in eggs of chum salmon (*Oncorhynchus keta*). Dev. Comp. Immunol..

[11] Ozaki H., Ohwaki M., Fukada T. (1983). Studies on lectins of amago (*Oncorhyncus rhodurus*) I. Amago ova lectin and its receptor on homologous macrophages. Dev. Comp. Immunol..

[12] Kudo S., Teshima C. (1991). Enzyme activities and antifungal action of fertilization envelope extract from fish eggs. J. Exp. Zool..

[13] Ovchinnikova T.V., Balandin S.V., Aleshina G.M., Tagaev A.A., Leonova Y.F., Krasnodembsky E.D., Men’shenin A.V., Kokryakov V.N. (2006). Aurelin, a novel antimicrobial peptide from jellyfish *Aurelia aurita* with structural features of defensins and channel-blocking toxins. Biochem. Biophys. Res. Commun..

[14] Hancock R.E.W., Scott M.G. (2000). The role of antimicrobial peptides in animal defenses. Proc. Natl. Acad. Sci. USA.

[15] Rocha J., Peixe L., Gomes N.C.M., Calado R. (2011). Cnidarians as a source of new marine bioactive compounds—An overview of the last decade and future steps for bioprospecting. Mar. Drugs.

[16] Stabili L., Licciano M., Pagliara P. (1994). Evidence of antibacterial and lysozyme-like activity in different planktonic larval stages of *Paracentrotus lividus*. Mar. Biol..

[17] Shugar D. (1952). The measurement of lysozyme activity and the ultra-violet inactivation of lysozyme. Biochim. Biophys. Acta.

[18] Canicatti C., Roch P. (1989). Studies on *Holothuria polii* (Echinodermata) antibacterial proteins. I. Evidence for and activity of a coelomocyte lysozyme. Experientia.

[19] Canicatti C. (1990). Protease activity in holothuria polh coelomic fluid and coelomocyte lysate. Comp. Biochem. Physiol..

[20] Stabili L., Schirosi R., Parisi M.G., Piraino S., Cammarata M. (2015). The mucus of *Actinia equina* (Anthozoa, Cnidaria): An unexplored resource for potential applicative purposes. Mar. Drugs.

[21] Leone A., Lecci R.M., Durante M., Meli F., Piraino S. (2015). The bright side of gelatinous blooms: Nutraceutical value and antioxidant properties of three Mediterranean jellyfish (Scyphozoa). Mar. Drugs.

[22] Zotti M., De Pascali S.A., Del Coco L., Migoni D., Carrozzo L., Mancinelli G., Fanizzi F.P. (2016). ^1^H NMR metabolomic profiling of the blue crab (*Callinectes sapidus*) from the Adriatic Sea (SE Italy): A comparison with warty crab (*Eriphia verrucosa*), and edible crab (*Cancer pagurus*). Food Chem..

[23] Tikunov A.P., Johnson C.B., Lee H., Stoskopf M.K., Macdonald J.M. (2010). Metabolomic investigations of American oysters using ^1^H-NMR spectroscopy. Mar. Drugs.

[24] De Pascali S.A., Del Coco L., Felline S., Mollo E., Terlizzi A., Fanizzi F.P. (2015). ^1^H NMR spectroscopy and MVA analysis of *Diplodus sargus* eating the exotic pest *Caulerpa cylindracea*. Mar. Drugs.

[25] Girelli C.R., De Pascali S.A., Del Coco L., Fanizzi F.P. (2016). Metabolic profile comparison of fruit juice from certified sweet cherry trees (*Prunus avium* L.) of Ferrovia and Giorgia cultivars: A preliminary study. Food Res. Int..

[26] Igarashi T., Aursand M., Hirata Y., Gribbestad I.S., Wada S., Nanaka M. (2000). Nondestructive quantitative determination of docosahexaenoic acid and n-3 fatty acids in fish oils by high-resolution ^1^H Nuclear Magnetic Resonance Spectroscopy. J. Am. Oil Chem. Soc..

[27] Sacchi R., Savarese M., Falcigno L., Giudicianni I., Paolillo L. (2008). Proton NMR of fish oils and lipids. Modern Magnetic Resonance.

[28] Shumilina E., Ciampa A., Capozzi F., Rustad T., Dikiy A. (2015). NMR approach for monitoring post-mortem changes in Atlantic salmon fillets stored at 0 and 4 °C. Food Chem..

[29] Nestor G., Bankefors J., Schlechtriem C., BräNnäS E., Pickova J., Sandström C. (2010). High-resolution ^1^H magic angle spinning nmr spectroscopy of intact arctic char (*Salvelinus alpinus*) muscle. quantitative analysis of n-3 fatty acids, EPA and DHA. J. Agric. Food Chem..

[30] Brotz L., Cheung W.W.L., Kleisner K., Pakhomov E., Pauly D. (2012). Increasing jellyfish populations: Trends in Large Marine Ecosystems. Hydrobiologia.

[31] Leone A., Lecci R.M., Durante M., Piraino S. (2013). Extract from the zooxanthellate jellyfish *Cotylorhiza tuberculata* modulates gap junction intercellular communication in human cell cultures. Mar. Drugs.

[32] Doyle T.K., Houghton J.D.R., McDevitt R., Davenport J., Hays G.C. (2007). The energy density of jellyfish: Estimates from bomb-calorimetry and proximate-composition. J. Exp. Mar. Biol. Ecol..

[33] Nicholson J.K., Foxall P.J.D., Spraul M., Farrant R.D., Lindon J.C. (1995). 750 MHz 1H and 1H-13C NMR Spectroscopy of Human Blood Plasma. Anal. Chem..

[34] Yu H., Li R., Liu S., Xing R.E., Chen X., Li P. (2014). Amino acid composition and nutritional quality of gonad from jellyfish *Rhopilema esculentum*. Biomed. Prev. Nutr..

[35] Khong N.M., Yusoff F.M., Jamilah B., Basri M., Maznah I., Chan K.W., Nishikawa J. (2016). Nutritional composition and total collagen content of three commercially important edible jellyfish. Food Chem..

[36] Imura K., Okada A. (1998). Amino acid metabolism in pediatric patients. Nutrition.

[37] Gülçin I. (2012). Antioxidant activity of food constituents: An overview. Arch. Toxicol..

[38] Zotti G. (2016). Open-source virtual archaeoastronomy. Mediterr. Archaeol. Archaeom..

[39] Mannina L., Sobolev A.P., Capitani D., Iaffaldano N., Rosato M.P., Ragni P., Reale A., Sorrentino E., D’Amico I., Coppola R. (2008). NMR metabolic profiling of organic and aqueous sea bass extracts: Implications in the discrimination of wild and cultured sea bass. Talanta.

[40] Berking S. (1997). 3 Hydrozoa Metamorphosis and Pattern Formation. Curr. Top. Dev. Biol..

[41] Siefker B., Kroiler M., Berking S. (2000). Induction of metamorphosis from the larval to the polyp stage is similar in Hydrozoa and a subgroup of Scyphozoa (Cnidaria, Semaeostomeae). Helgol. Mar. Res..

[42] Yancey P.H. (2005). Organic osmolytes as compatible, metabolic and counteracting cytoprotectants in high osmolarity and other stresses. J. Exp. Biol..

[43] Seibel B.A., Walsh P.J. (2002). Trimethylamine oxide accumulation in marine animals: Relationship to acylglycerol storage. J. Exp. Biol..

[44] Abdullah A., Nurjanah N., Hidayat T., Aji D.U. (2015). Fatty Acid Profile of Jellyfish (*Aurelia aurita*) as a Source Raw Material of Aquatic Result Rich Beneft. Int. J. Chem. Biol. Sci..

[45] Besnard J.Y. (1988). Etude des Constituents Lipidiques Dans la Gonade Femelle et les Larves de *Pecten maximus* L. (Mollusque Lamellibranche). Ph.D. Dissertation.

[46] Izquierdo M.S., Fernández-Palacios H., Tacon A.G.J. (2001). Effect of broodstock nutrition on reproductive performance of fish. Aquaculture.

[47] Tulli F., Tibaldi E. (1997). Changes in amino acids and essential fatty acids during early larval rearing of dentex. Aquac. Int..

[48] Miliou H., Fintikaki M., Tzitzinakis M., Kountouris T., Verriopoulos G. (2006). Fatty acid composition of the common octopus, *Octopus vulgaris*, in relation to rearing temperature and body weight. Aquaculture.

[49] Xu Y., Cleary L.J., Byrne J.H. (1994). Identification and characterization of pleural neurons that inhibit tail sensory neurons and motor neurons in Aplysia: Correlation with FMRFamide immunoreactivity. J. Neurosci..

[50] Stanley-Samuelson D.W. (1987). Physiological roles of prostaglandins and other eicosanoids in invertebrates. Biol. Bull..

[51] Nichols P.D., Danaher K.T., Koslow J.A. (2003). Occurrence of High Levels of Tetracosahexaenoic Acid in the Jellyfish *Aurelia* sp.. Lipids.

[52] Song C., Zhao S. (2007). Omega-3 fatty acid eicosapentaenoic acid. A new treatment for psychiatric and neurodegenerative diseases: A review of clinical investigations. Expert Opin. Investig. Drugs.

[53] Song C., Phillips A.G., Leonard B.E. (2003). Interleukin-1β enhances conditioned fear memory but impairs spatial learning in rats: Possible involvement of glucocorticoids and monoamine function in the amygdala and hippocampus. Eur. J. Neurosci..

[54] Marquis C.P., Baird A.H., De Nys R., Holmström C., Koziumi N. (2005). An evaluation of the antimicrobial properties of the eggs of 11 species of scleractinian corals. Coral Reefs.

[55] Kelman D., Kushmaro A., Loya Y., Kashman Y., Benayahu Y. (1998). Antimicrobial activity of a Red Sea soft coral, *Parerythropodium fulvum fulvum*: Reproductive and developmental considerations. Mar. Ecol. Prog. Ser..

[56] Gunthorpe L., Cameron A.M. (1990). Widespread but variable toxicity in scleractinian corals. Toxicon.

[57] Gunthorpe L., Cameron A.M. (1990). Intracolonial variation in toxicity in scleractinian corals. Toxicon.

[58] Bowden B., Coll J., Willis R. Some chemical aspects of spawning in alcyonacean corals. Proceedings of the Fifth International Coral Reef Congress.

[59] Baird A.H., Pratchett M.S., Gibson D.J., Koziumi N., Marquis C.P. (2001). Variable palatability of coral eggs to a planktivorous fish. Mar. Freshw. Res..

[60] Fusetani N., Toyoda T., Asai N., Matsunaga S., Maruyama T. (1996). Montiporic acids A and B, cytotoxic and antimicrobial polyacetylene carboxylic acids from eggs of the scleractinian coral *Montipora digitata*. J. Nat. Prod..

[61] Lesser M.P., Stochaj W.R., Tapley D.W., Shick J.M. (1995). Bleaching in coral reef anthozoans: Effects of irradiance, ultraviolet radiation, and temperature on the activities of protective enzymes against active oxygen. Coral Reefs.

[62] Leclerc M., Rinkevich B., Muller W.E.G. (1996). Humoral factors in marine invertebrates. Molecular and Subcellular Biology: Invertebrate Immunology.

[63] Stryer L. (2013). Biochemie.

[64] Jielian W., Baoqing H., Chungen W., Peipei Y. (2017). Characterization and roles of lysozyme in molluscs. ISJ.

[65] Chang K.Y., Carr C.W. (1917). Studies on the structure and function of lysozyme: I. The effect of pH and cation concentration on lysozyme activity. Biochim. Biophys. Acta.

[66] Davies R.C., Neuberger A., Wilson B.M. (1969). The dependence of lysozyme activity on pH and ionic strength. Biochim. Biophys. Acta.

[67] Labro M.T. (1997). The prohost effect of antimicrobial agents as a predictor of clinical outcome. J. Chemother..

[68] SeGall G.K., Lennarz W.J. (1979). Chemical characterization of the component of the jelly coat from sea urchin eggs responsible for induction of the acrosome reaction. Dev. Biol..

[69] Slattery M., McClintock J.B. (1995). Population structure and feeding deterrence in three shallow-water antarctic soft corals. Mar. Biol..

[70] Dubois M., Gille K.A., Hamilton J.K., Rebers P.A., Smith F. (1956). Colorimetric method for determination of sugars and related substances. Anal. Chem..

[71] Lowry O.H., Rosebrough N.J., Farr A.L., Randall R.J. (1951). Protein measurement with the Folin phenol reagent [Folin-Ciocalteu reagent]. J. Biol. Chem..

[72] Barnes H., Blackstock J. (1973). Estimation of lipids in marine animals and tissues: Detailed investigation of the sulphophosphovanilun method for “total” lipids. J. Exp. Mar. Bio. Ecol..

[73] Jacobs D.S., Kasten B.L., Demott W.R. (1991). Laboratory Test Handbook, 2nd ed. J. Intraven. Nurs..

[74] Bucolo G., David H. (1973). Quantitative determination of serum triglycerides by the use of enzymes. Clin. Chem..

[75] Bligh E.G., Dyer W.J. (1959). A rapid method of total lipid extraction and purification. Can. J. Biochem. Physiol..

[76] Wu H., Southam A.D., Hines A., Viant M.R. (2008). High-throughput tissue extraction protocol for NMR- and MS-based metabolomics. Anal. Biochem..

[77] Moreira-Ferro C.K., Daffre S., James A.A., Marinotti O. (1998). A lysozyme in the salivary glands of the malaria vector *Anopheles darlingi*. Insect Mol. Biol..

[78] Daffre S., Kylsten P., Samakovlis C., Hultmark D. (1994). The lysozyme locus in *Drosophila melanogaster*: An expanded gene family adapted for expression in the digestive tract. Mol. Gen. Genet..

[79] Smith L.C., Arizza V., Barela Hudgell M.A., Barone G., Bodnar A.G., Buckley K.M., Cunsolo V., Dheilly N.M., Franchi N., Fugmann S.D., Cooper E.L. (2018). Echinodermata: The Complex Immune System in Echinoderms. Advances in Comparative Immunology.

[80] Anderson M.J. (2001). A new method for non-parametric multivariate analysis of variance. Austral Ecol..

[81] Anderson M., Braak C. (2003). Ter Permutation tests for multi-factorial analysis of variance. J. Stat. Comput. Simul..

[82] Clarke K.R. (1993). Non-parametric multivariate analyses of changes in community structure. Aust. J. Ecol..

